# Pediculated myxoma from atrial septum invading atria and biventricular inlets

**DOI:** 10.5935/1678-9741.20150048

**Published:** 2015

**Authors:** Camila Caetano Cardoso, Ulisses Alexandre Croti, Carlos Henrique De Marchi, Airton Camacho Moscardini

**Affiliations:** 1 Serviço de Cardiologia e Cirurgia Cardiovascular Pediátrica de São José do Rio Preto - Hospital da Criança e Maternidade de São José do Rio Preto and Faculdade de Medicina de São José do Rio Preto (FAMERP), São José do Rio Preto, SP, Brazil.

**Keywords:** Myxoma, Heart Murmurs, Dyspnea

**Table:** 

**Abbreviations, acronyms & symbols**	
CPB	Cardiopulmonary bypass

## CLINICAL DATA

A 7 years and 10 months old male child, 25 kg, born and raised in São José do Rio Preto, SP, referred for heart murmur and fatigue investigation.

Dyspneic for three months and progressive worsening associated to sporadic dorsal region pain upon moderate exertion. Three days earlier he presented postprandial vomiting, loss of appetite and worsening of general condition.

Upon physical examination the patient was in regular state: pale, hydrated, eupneic and afebrile. Regular heart rhythm with systolic murmur 4 + / 6 + and tachycardia. Clear lung sounds. Distended and painful abdomen upon palpation, along with hepatomegaly (liver palpable at 2.36 inches from the right costal margin). Blood pressure and heart rate were normal in all four limbs and without edema.

## ELECTROCARDIOGRAM

Sinus rhythm with a heart rate of 122 beats/min, QRS axis + 30º and PR interval of 0.12 s. Overload in both atria without ventricular overload. Ventricular repolarization unchanged.

## RADIOGRAPHY

Visceral *situs solitus* in levocardia. Increased cardiac area with a cardiothoracic index of 0.65 and pulmonary vasculature within normal limits.

## ECHOCARDIOGRAM

*Situs solitus* in levocardia, all connections were concordant. Significant dilation of both atria with moderate mitral valve regurgitation and important tricuspid valve regurgitation. Moderate pericardial effusion.

**Fig. 1:**
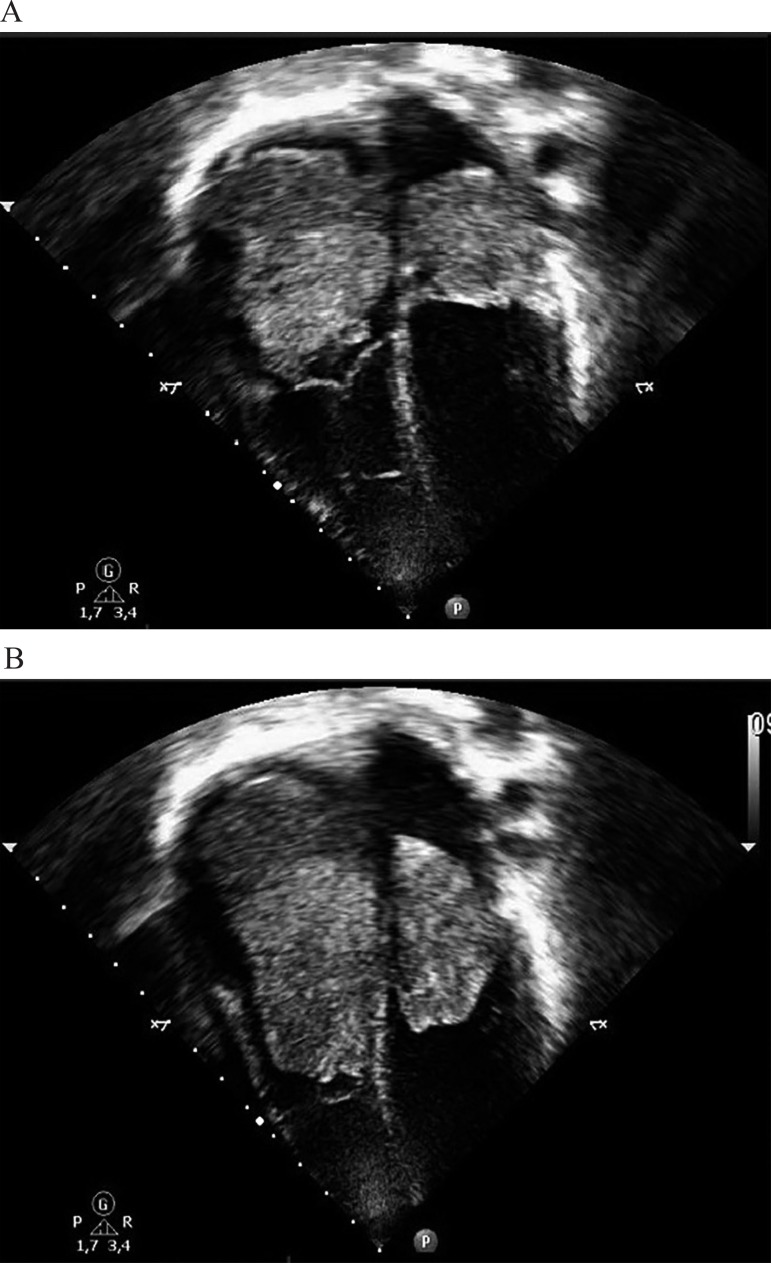
A. Two-dimensional echocardiogram preoperatively showing the masses within the atria. The largest mass is located in the right atrium and occupies all of its cavity. It measures 2.75 x 1.57 inches in its greatest diameter. The mass in the left atrium measuring 1.77 x 1.18 inches; B. Invagination of the masses to the ventricles interior during diastole.

Presence of large pediculated, lobed and homogeneous mass originating from the atrial septum occupying both right and left atria and projecting into the biventricular inlet tract during diastole, causing blood flow obstruction. Normal ejection fraction (Figures 1A and 1B).

## DIAGNOSIS

Myxomas represent around half of all heart tumors and may be associated with dominant family autosomal syndromes. The majority of them affects the left atrium, but can be present in other sites. The main differential diagnosis is rhabdomyoma^[1]^.

The suggestive clinical status of low cardiac output, altered cardiac auscultation and the presence of two intracardiac tumor mass on echocardiography were fundamental for the diagnosis and surgical resection indication^[1,2]^.

**Fig. 2:**
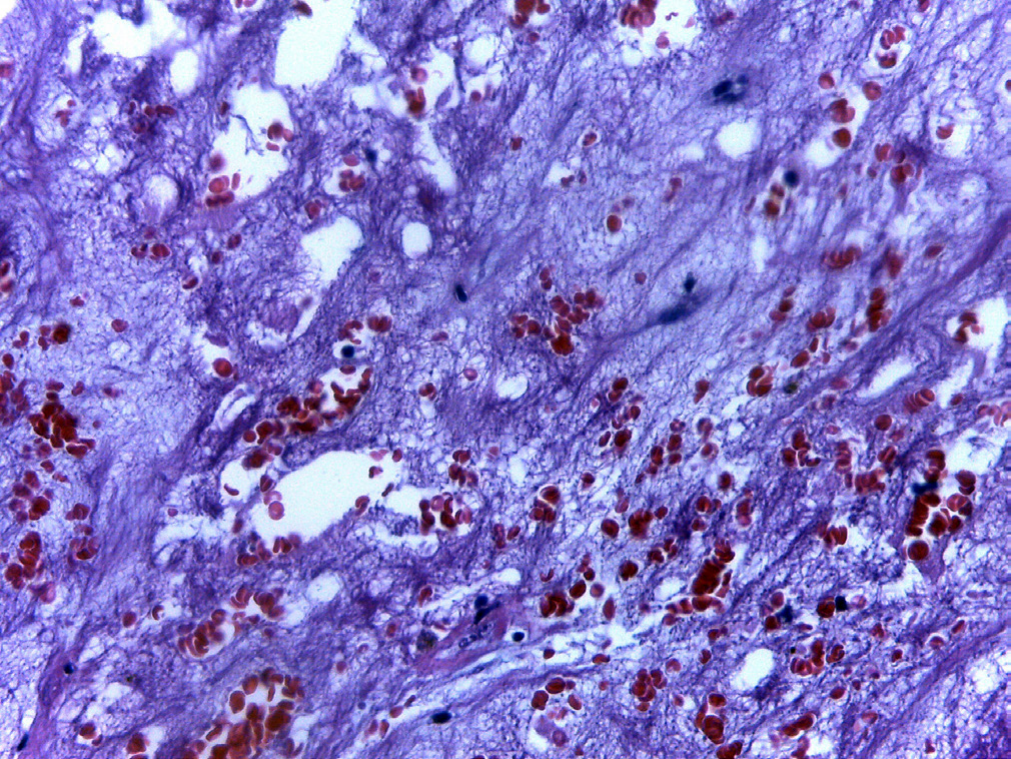
Microscopically round, polygonal, or stellate cells are seen surrounded by abundant loose stroma rich in acid mucopolysaccharides. Myxoid stroma with recent and late hemorrhagic areas with hemosiderin pigments.

The radiological exam used for this diagnosis was the echocardiogram because it is a non-invasive exam and has an excellent sensitive. The histologic diagnosis was confirmed by pathological examination after operation, as it is shown in Figure 2^[3,4]^.

## OPERATION

Median sternotomy found a greatly increased right atrium. Heparinization at 4mg/kg and careful aorta and vena cava cannulation were performed to avoid embolization. Cardiopulmonary bypass (CPB) was initiated, antegrade blood cardioplegia, hypothermic at 39ºF and intermittent every 20 minutes.

Right atrium was opened and large gelatinous mass was found, darkened and ocher colored. It was pulled gently releasing the entire right ventricular inlet and noting that there was no adhesion of the mass to the right atrial or ventricular walls, just fixed to the atrial septum. It was opted for resection of the atrial septum since the additional tests showed presence of mass also occluding the left side. After opening the atrial septum it was observed that the tumor obstructing both sides originated from the same site.

**Fig. 3:**
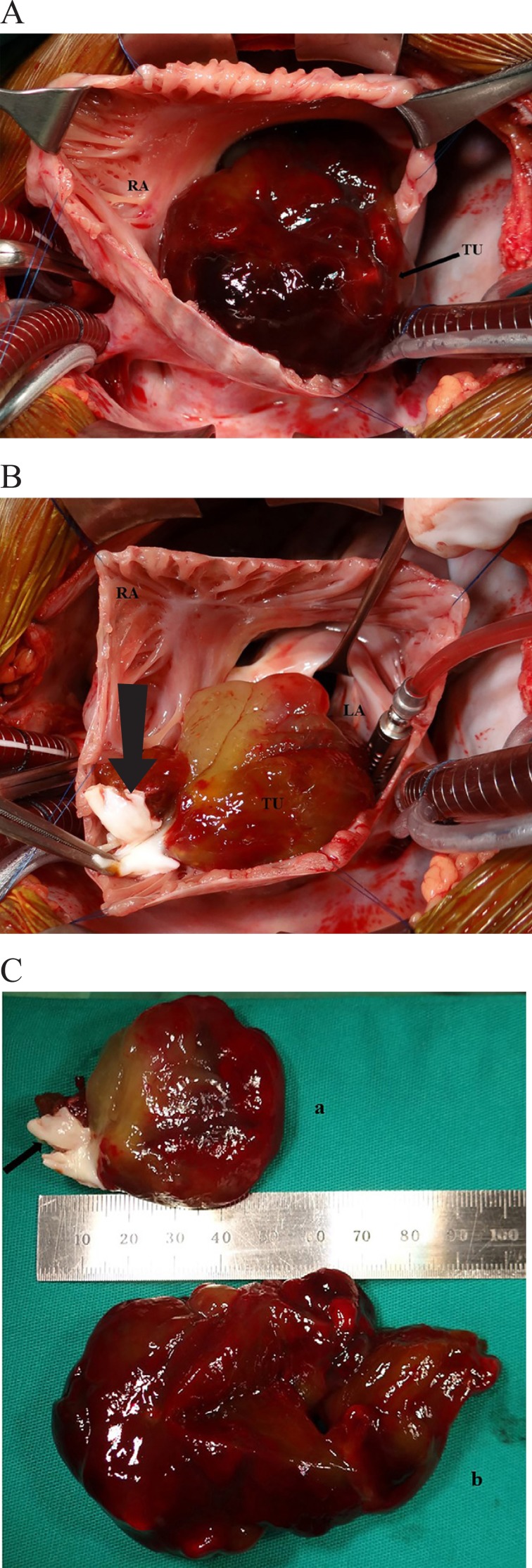
A. Pediculated tumor in the atrial septum (arrow) removed from right atrium and right ventricular inlet. Note that there was no adhesion to the cavities; B. Atrial septum resected with the pediculated tumor and obstructing the left atrium and the left ventricle inlet. The arrow indicates the remnants of the tumor to the right of the atrial septum featuring both cavities affected by the same mass; C. Resected tumor diameters to the left (a) and to the right (b). In the atrial septal remnant (arrow) can be observed the presence of the tumor witch occupied the right and left atria.

The atrial septum was completely resected along with the tumor, which also showed no adhesions in the left cavities, subsequently reconstructed with bovine Braile Biomédica^®^ pericardial patch in a conventional way (Figures 3A, 3B and 3C).

The CPB time was of 50 minutes and myocardial ischemia of 34 minutes at 93ºF.

The postoperative period was uneventful with hospital discharge after six days of hospitalization.

**Table:** 

**Authors’ roles & responsibilities**	
CCC	Analysis and/or data interpretation; conception and design study; final manuscript approval; manuscript writing or critical review of its content
UAC	Analysis and/or data interpretation; conception and design study; final manuscript approval; manuscript writing or critical review of its content; realization of operations and/or trials; statistical analysis
CHDM	Analysis and/or data interpretation; conception and design study; final manuscript approval; manuscript writing or critical review of its content
ACM	Analysis and/or data interpretation; conception and design study; final manuscript approval

## References

[1] Beroukhim RS, Prakash A, Buechel ER, Cava JR, Dorfman AL, Festa P (2011). Characterization of cardiac tumors in children by cardiovascular
magnetic resonance imaging: a multicenter experience. J Am Coll Cardiol.

[2] Padalino MA, Vida VL, Boccuzzo G, Tonello M, Sarris GE, Berggren H (2012). Surgery for primary cardiac tumors in children: early and late
results in a multicenter European Congenital Heart Surgeons Association
study. Circulation.

[3] Pinede L, Duhaut P, Loire R (2001). Clinical presentation of left atrial cardiac myxoma. A series of
112 consecutive cases. Medicine (Baltimore).

[4] Croti UA, Braile DM, Souza AS, Cury PM (2008). Right ventricle and tricuspid valve myxoma. Rev Bras Cir Cardiovasc.

